# A Survey of Oral Health-Related Quality of Life for Adults with Cerebral Palsy in Australia

**DOI:** 10.3390/dj13090407

**Published:** 2025-09-04

**Authors:** Karen Lansdown, Kim Bulkeley, Margaret McGrath, Michelle Irving, Claudia Zagreanu, Hayley Smithers-Sheedy

**Affiliations:** 1 Department of Oral Health, School of Acute and Primary Health, Faculty of Health and Environmental Sciences, Auckland University of Technology, Auckland 0627, New Zealand; claudia.zagreanu@aut.ac.nz; 2 School of Dentistry, Faculty of Medicine and Health, The University of Sydney, Sydney, NSW 2006, Australia; 3 Sydney School of Health Sciences, Faculty of Medicine and Health, The University of Sydney, Sydney, NSW 2006, Australia; kim.bulkeley@sydney.edu.au; 4 School of Clinical Therapies, College of Medicine and Health, University College Cork, T12 EK59 Cork, Ireland; margaretmcgrath@ucc.ie; 5 Office for Health and Medical Research, New South Wales Ministry of Health, Sydney, NSW 1590, Australia; michelle.irving@health.nsw.gov.au; 6 Cerebral Palsy Alliance Research Institute, Specialty of Child & Adolescent Health, Sydney Medical School, Faculty of Medicine & Health, The University of Sydney, Sydney, NSW 2050, Australia; hsmitherssheedy@cerebralpalsy.org.au

**Keywords:** cerebral palsy, disability, OHRQoL, dental care and services, OHIP-14

## Abstract

**Objective:** Our aim was to investigate the oral health-related quality of life (OHRQoL) and dental care experiences of adults with Cerebral Palsy (CP). **Methods:** In 2023, adults with CP and their caregivers from four Australian states completed questionnaires, including the Oral Health Impact Profile-14 (OHIP-14). Non-parametric tests were conducted to analyze associations between demographic and CP-related variables and OHRQoL. **Results:** A total of 69 respondents participated, including *n* = 22 adults with CP and *n* = 47 caregivers of adults with CP. Most adults with CP were diagnosed with a spastic motor type (46/69, 66%), with bilateral spasticity being the most common (30/46, 65%). The mean OHIP-14 score was 10.3 ± 9.3 (mean ± SD). Nearly 70% reported challenges cleaning their teeth, over 25% lacked a dentist, more than 60% found dental exams challenging, and nearly 50% required specialized dental care. In bivariate analysis, OHIP-14 was associated with daily oral care routines (*p* = 0.012) and “simple dental check-up” (*p* = 0.017). There was a statistically significant relationship between socio-economic status and scores for the handicap dimension (*p* = 0.040). Higher OHIP-14 scores were associated with greater levels of impairment regarding gross motor (*p* = 0.199), manual functioning limitations (*p* = 0.001), speech (*p* = 0.123), and communication function scales (*p* = 0.319). **Conclusion:** Adults with CP reported challenges participating in and maintaining oral health and accessing dental care, influenced by physical, functional, and socio-economic factors. These findings indicate the need for inclusive care and strategies to support access to services.

## 1. Introduction

Cerebral palsy (CP) encompasses a group of movement disorders resulting from injury or maldevelopment of the developing brain. Whilst motor disorders are lifelong and permanent, they can change over time [1]. People with CP often experience associated conditions such as intellectual disabilities and epilepsy [2]. The functional motor limitations and associated impairments of CP can significantly impact daily functioning, overall well-being, and quality of life [3,4,5].

Oral health plays a significant role in well-being, quality of life, and social participation [6]. Adults with CP can experience challenges related to oral health due to factors such as muscle spasticity, dysphagia, and difficulties in maintaining oral hygiene, leading to a greater risk of oral health problems, including dental caries, periodontal disease, and malocclusion [7,8,9,10]. These oral health issues can affect the ability to eat and/or speak and can result in feelings of embarrassment or frustration with self-image. Past dental experiences can influence how people with CP perceive expectations of dental care and the treatment they receive [11].

A 2023 Australian Senate Select Committee highlighted significant barriers to accessible dental care for Australians with disabilities, revealing a shortage of specialized services, with some geographical areas lacking any dental specialists [12]. Research on dental care access for adults with CP worldwide remains limited; the existing literature, to date, has focused primarily on pediatric populations, with a recent scoping review finding a significant gap in the available evidence related to dental services for people with CP [10].

Understanding the OHRQoL for people with CP is essential so that services can be tailored to meet needs; however, Australian-specific data on the oral health challenges of adults with CP remains scarce. This study addresses this knowledge gap by (i) exploring OHRQoL among adults with CP and (ii) describing the dental care experiences of adults with CP.

Therefore, this study aimed to evaluate the impact of OHRQoL and report on the dental care experiences of adults with CP.

## 2. Materials and Methods

### 2.1. Study Design and Ethics

This cross-sectional study collected data via an online survey. The work was guided by three research partners with lived experiences of CP. Ethical approval was obtained by The University of Sydney Human Research Committee, [2023/127] on the 13 April 2023, and governance approvals were received from the Cerebral Palsy Alliance [2023_04_01] on the 22 May 2023 and Choice Passion Life [CPL-2023-001] on the 11 July 2023.

### 2.2. Participants

Adults with CP or their caregivers aged 18 years and over were recruited from CP registers in New South Wales/Australian Capital Territory, Queensland, and Victoria. Invitations were sent via email, and newsletters were received between May and August 2023, with reminders sent after eight weeks by two of the CP registers. Inclusion criteria included proficiency in English, functional vision, and access to a computer or device. Participation was voluntary and anonymous.

### 2.3. Data Collection

Participant data were collected via Research Electronic Data Capture (REDCap) [13], which is a secure, web-based platform hosted by The University of Sydney, designed to ensure data security and privacy [14]. Duplicate responses were identified using initials (first, middle, and last) and postcodes. The survey included demographic data and CP clinical details. Country of birth was recorded as Australia or overseas, and postcodes were classified according to the Australian Statistical Geography Standard (major cities, inner regional, outer regional, and remote/very remote areas) [15]. Socio-economic status was assessed using the Socio-Economic Indexes for Areas (SEIFA) with SEIFA quintiles ranging from 1 = most disadvantaged to 5 = most advantaged [16]. Information on CP included motor type (spastic, dyskinetic, or ataxic) and spastic topography (unilateral or bilateral). Functional mobility was classified using the Gross Motor Function Classification System (GMFCS), grouped into “Ambulant” (levels I-II) and “Supported Mobility” groups (levels III-V) [17]. Manual ability was categorized using the Manual Ability Classification System (MACS) into “Handles objects” (I-II) and “Requires support to handle objects” (III-V) [18]. Speech intelligibility was categorized using the Viking Speech Scale (VSS), divided into two categories: “speech understandable to all listeners” (I-II) and “speech not understandable to all listeners” (III-IV) [19]. Functional communication was classified using the Communication Function Classification System (CFCS) and categorized into two levels: “effectively communicates with most partners” (I-II) and “difficulty with communicating effectively with some or all partners” (III-V) [20]. Comorbidities, including hearing impairments, epilepsy, and the use of anti-seizure medications, were also documented.

In the first section of the survey, participants were asked to complete the validated short form of the English version of the Oral Health Impact Profile-14 (OHIP-14) survey, which evaluates individuals’ perceptions of how oral conditions affect their well-being. This tool covers seven dimensions related to teeth, mouth, or false teeth [21]. Dimensions include functional limitation (e.g., trouble pronouncing any words or worsening sense of taste); physical pain (e.g., life, in general, has become less satisfying or individual is unable to function); psychological discomfort (e.g., being self-conscious or feeling tense); physical disability (e.g., unsatisfactory diet or interrupted meals); psychological disability (e.g., finds it difficult to relax or is embarrassed); social disability (e.g., a bit irritable with other people or difficulty performing jobs); and handicap dimension (e.g., life, in general, was less satisfying or the individual has been totally unable to function). The OHIP-14 uses a 5-point Likert scale to assess the frequency of impacts over the last 12 months. Responses were coded 4 = “very often”; 3 = “fairly often”; 2 = “occasionally”; 1 = “hardly ever”; and 0 = “never”. Summary measures included mean scores and the percentage of items reported as “fairly often” or “very often.” The total OHIP-14 score (0 to 56) is the sum of individual item scores, with lower scores indicating fewer oral health impacts [21].

The second section of the survey focused on participants’ experiences of oral health, including oral hygiene routines, access to dental care, the impact of CP on dental care, home visits, urgent care, and complex surgeries. Questions in this section were adapted from a questionnaire by Liu et al. 2022, [22] with the term “disability” replaced by “CP” throughout.

### 2.4. Statistical Analysis

Data were analyzed using SPSS Version 29 [23]. Descriptive statistics were used to summarize clinical and demographic information. Participants with missing demographic, clinical, or dental care experience data were still included in the analysis. OHIP-14 questionnaires with more than five missing items (including “don’t know” answers) were excluded. For the questionnaires with ≤5 items, a regression procedure was used to impute the missing data. Internal consistency of the OHIP-14 scale was evaluated using Cronbach’s coefficient alpha with a minimum required sample size of *n* = 38 [24,25]. The magnitude of Cronbach’s alpha was judged in accordance with published guidelines; a coefficient ≥ 0.70 was considered to indicate satisfactory internal consistency [26]. The construct validity was assessed by examining the relationship between the overall OHIP-14 score and the self-reported daily oral health routine (“fairly straightforward”, “a bit of a challenge”, and “extremely difficult”). Due to the lack of prior studies, we were unable to estimate the sample size required to investigate the relationship between the independent (demographic and CP-related) and dependent (OHIP-14 score) variables. All independent variables were categorical (nominal or ordinal), whereas the independent variable was continuous. Normality and homogeneity of the dependent variable were assessed using the Shapiro–Wilk and Levene tests. Non-parametric tests [Mann–Whitney U (MWU) and Kruskal–Wallis H (KWH) tests] were used to compare the statistical significance of the difference between the OHIP-14 scores of categorical variables. Variables that obtained values of *p* ≤ 0.20 in bivariate analysis were added to a multiple regression analysis. Statistical significance was set as *p* < 0.05. Reporting adhered to the Consensus-Based Checklist for Reporting of Survey Studies (CROSS) [27].

## 3. Results

Seventy-four surveys were completed. After incomplete and duplicate responses were removed, 69 surveys (22 self-reports and 47 caregiver proxy-reports) were analyzed. Just over half of the *n* = 69 adults with CP were male, and the majority (75%) resided in major cities. Spasticity was the most common motor type of CP (67%); of those with spasticity, 65% had bilateral spastic CP. A total of 67% of participants required assistive equipment for mobility (GMFCS levels III-V), and 71% experienced difficulties in handling objects (MACS levels III-V). Half had epilepsy, with 40% of those with epilepsy having been prescribed anti-seizure medication. Table 1 presents demographic information and clinical characteristics.

The OHIP-14 scale showed high internal consistency of Cronbach’s alpha = 0.902 [26]. The analysis of the inter-item correlations matrix revealed positive correlations between all items. The corrected item–total correlations ranged from 0.387 (“Have you had painful aching in your mouth?”) to 0.735 (“Have you had to interrupt meals because of problems with your teeth or mouth?”), which was above the recommended minimum value of 0.20 to include an item on a scale [26]. The removal of one item at a time resulted in lower Cronbach’s alpha values compared to the original values obtained, supporting the inclusion of all the items (Table 2). The construct validity was tested by evaluating the relationship of OHIP-14 and its dimensions with the self-perceived oral health daily routine. There was a statistically significant positive correlation between daily routine and the OHIP-14 total score and five of its dimensions (Table 3).

**Figure 1:**
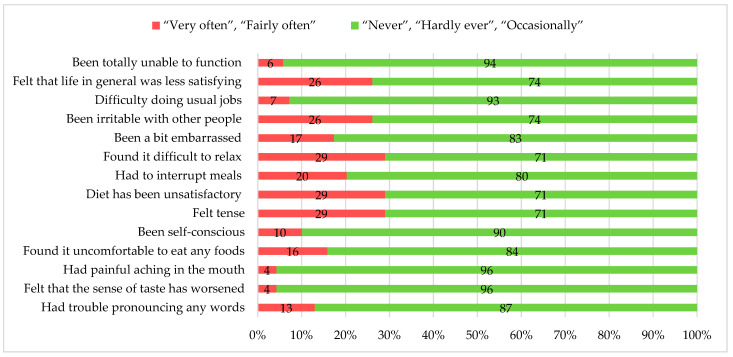
Oral health impact profile participant responses by question.

Scores on the scale ranged from 0 to 56, with lower scores indicating better OHRQoL. The mean OHIP-14 score was 10.3 ± 9.3. The range of individual responses varied widely. Notably, 13% of this group reported difficulty pronouncing words; nearly 16% found eating uncomfortable; and 29% felt tense because of problems with their teeth, mouth, or false teeth, with the same percentage reporting an unsatisfactory diet and difficulty relaxing. Oral health also impacted social interactions, with more than a quarter of this group (26%) reporting that they experience irritability with others and lower life satisfaction due to oral health issues (Figure 1).

No statistically significant differences in OHRQoL were found by age, sex, remoteness, type of CP, or hearing ability. The MWU test showed that the median OHIP-14 score for males and females was not statistically significantly different (*p* = 0.673); however, males scored higher than females in terms of functional limitation, psychological discomfort, and psychological disability, while females scored higher in terms of physical pain and social disability (Table 4). Adults with CP living in the most disadvantaged areas (SEIFA Quintiles 1 and 2) had a greater risk of oral health impacts (OHIP) in the OHIP handicap dimension (e.g., life, in general, has become less satisfying or the individual is unable to function because of problems with their teeth, mouth, or false teeth) than those living in the most advantaged areas (*p* = 0.040). Individuals with greater functional motor limitations had greater oral health impacts. For example, adults with CP described as GMFCS levels III-V (requiring use of assistive mobility devices at some or all times), reported greater risk for oral health impacts in the OHIP physical pain dimension (e.g., life, in general, has become less satisfying or the individual is unable to function), than those described as GMFCS levels I-II (who do not require the use of assistive mobility devices) (*p* = 0.037) (Table 4). Similarly, participants with MACS levels III-V (indicating a difficulty or inability to handle objects), experienced a significantly greater risk for oral health impact for the OHIP functional limitation (*p* = 0.015), OHIP physical pain (*p* = 0.009), OHIP psychological discomfort (e.g., self-conscious or feeling tense) (*p* = 0.037), and OHIP psychological disability dimensions (e.g., finds it difficult to relax or is embarrassed) (*p* = 0.013) compared to those participants with MACS levels I-II (with a greater ability to handle objects) (Table 4). Participants with VSS, levels (III-IV), which reflect more significant speech difficulties, reported a greater risk for oral health impacts in the OHIP functional limitation (*p* = 0.024), with an average score of 2.2 ± 2.3 compared to 0.9 ± 1.1 for those at levels I-II.

Participants with CFCS Levels III-V (reflecting difficulty in functional communication) experienced a greater risk for oral health impacts in the OHIP physical disability dimension (e.g., unsatisfactory diet or interrupted meals), compared to those participants with CFCS Levels I-II (*p* = 0.033) (Table 4). Participants with epilepsy who were taking anti-seizure medication experienced a significantly greater risk for oral health impacts in the OHIP dimensions of functional limitation (*p* = 0.040), physical disability (*p* = 0.030), psychological disability (*p* = 0.034), and social disability (*p* = 0.045).

When comparing direct versus proxy reports, it was also noted that caregivers who completed the surveys by proxy reported a greater risk for oral health impacts in the OHIP functional limitation dimension (e.g., trouble pronouncing words or worsened taste) (*p* = 0.020) compared with adults with CP who were able to self-report. Table 4 presents these results.

### Dental Care Experiences

Most adults with CP (70%) reported difficulty cleaning their teeth, and over a quarter of participants (26%) did not have a dentist. More than 60% of participants indicated that CP negatively impacted their participation in dental examinations. Whilst nearly half of participants (48%) could access a dental assessment and scale and clean without specialized services, more than a third (36%) required day surgery/hospital care for services. Only two participants reported having a dental professional come to their home for examination or treatment. Table 5 presents these results.

Participants who reported extreme difficulty with cleaning their teeth had a greater risk for oral health impacts (*p* = 0.012) overall and across four out of the seven OHIP dimensions, including functional limitation (*p* = 0.013), physical pain (*p* = 0.021), physical disability (*p* = 0.007), and social disability (*p* = 0.026) (Table 6). In contrast, participants who could access a simple examination and scale and clean without needing special arrangements had a lower overall OHIP-14 score (*p* = 0.017) and reported less impact for the OHIP functional limitation (*p* = 0.007), physical pain (*p* = 0.039), and physical disability (*p* = 0.006) dimensions (Table 6). Those who did not require special arrangements for dental care reported lower overall OHIP-14 scores (*p* = 0.034), suggesting that easier access to dental care is linked to better OHRQoL. Table 6 presents these relationships.

Starting with six predictors, a weighted least squares (WLSs) regression analysis was run to predict the OHIP-14 score from the MACS, GMFCS, VSS, oral health daily routine, “simple dental check-up clean, no special requirements needed”, and “special arrangements were needed to attend the local practice”, accounting for heteroscedasticity. The weights were calculated as the inverse of the squared residuals from an initial standard regression analysis. The model statistically significantly predicted the OHIP-14 score, *F*(4,61) = 4.206, and *p*-value < 0.001. The MACS was statistically significantly associated with the OHIP-14 score (β = 0.328, SE B = 2.331, *p* = 0.024, 95% CI [0.750, 10.073]), suggesting that participants with MACS levels III-IV have higher OHIP-14 scores. Regression coefficients and standard errors can be found in Table 7.

## 4. Discussion

This is the first Australian study to explore OHRQoL in adults with CP and included both self-reports from adults with CP and proxy reports by caregivers. The results confirm that the OHIP-14 scale is a reliable and valid instrument to measure OHRQoL for adults with CP. OHIP scores varied significantly, reflecting the heterogeneity of clinical and functional characteristics of CP and the variability of perceived impacts on oral health.

In this study, sex and age did not have a significant association with the OHIP-14 score or its dimensions, as shown in bivariate analyses. This is in accordance with other investigations in which age and sex were found to have no significant association with OHRQoL [28].

In this study, adults with CP from disadvantaged areas experienced significantly greater oral health impacts, including life dissatisfaction, compared to those from the most advantaged quintiles. These findings are consistent with the existing literature that links lower SES with reduced health care access and poorer health outcomes [29]. The intersection between socio-economic challenges and complex oral health needs in adults with CP emphasizes the urgent need for national oral health policy recommendations that promote equitable access to dental care and address persistent disparities, particularly in this underserved group.

Adults with CP, particularly those with greater functional motor limitations (GMFCS III-V), were shown to experience oral health challenges that impact both their quality of life and daily functionality. Importantly, it was noted that individuals who had more difficulty handling objects (MACS III-V) reported greater oral health impacts across many OHIP scale items, including speech clarity, taste perception, physical discomfort during eating, self-consciousness, tension, and difficulty relaxing and/or feelings of embarrassment. Findings from the WLS regression demonstrate that people with MACS III-V have higher total OHIP-14 scores and therefore lower OHRQoL. This reinforces the importance of designing treatment plans that go beyond addressing immediate oral health needs but that recognize the broader impacts of clinical factors (in particular, MACS, CFCS, and VSS) and how these may influence OHRQoL.

Similarly, adults with CP who had greater speech and communication challenges (VSS III-IV and CFCS III-V) reported being impacted across multiple OHIP-14 domains, likely reflecting broader neuromuscular and feeding difficulties [30,31]. It is important for dental professionals to be better informed about the link between communication, feeding, and oral health in people with CP. Adopting an interdisciplinary approach that integrates speech and occupational therapists in the development of individualized oral health care plans may significantly enhance the OHRQoL of adults with CP [32].

This study has shown that individuals who have epilepsy and report taking anti-seizure medication have a greater risk for oral health impacts. Anti-seizure medications are well-known to have potential effects such as gingival overgrowth, xerostomia, and/or an increased risk of dental caries [33]. Adults with CP and their caregivers need further education on the gingival effects of certain medications as part of individualized oral health plans to ensure that both oral and systemic needs are addressed.

In terms of access to oral health care, this study’s findings align with the findings of Liu et al. 2022 [22] and a recent Australian study by Lansdown et al. 2025 [32] both of which have identified ongoing barriers to oral health care for individuals with disabilities across their lifespan. Adults with CP and their caregivers report experiencing barriers to dental care and find dental participation challenging. Participants in this study who found their daily oral care routines “extremely difficult” commonly reported greater functional limitations, greater physical pain, disability, and social challenges, reflecting their complex needs. Traditional clinic-based models may not adequately meet the needs of adults with CP, particularly for those who require supportive mobility. Dental professionals need to consider alternative service delivery models that may include home and community-based care, mobile dental clinics, and tele dentistry to improve access to care and, more importantly, the continuity of care to reduce unmet dental needs and improve OHRQoL.

Adults with CP more frequently require sedation and rely on hospital or day surgery facilities for dental procedures. These findings support the need for a structured referral framework and targeted professional development opportunities to equip dental professionals with the competencies and confidence necessary to manage complex cases, facilitate timely access to specialist care, and improve treatment outcomes [34]. These findings emphasize the need for patient-centered care frameworks, which are adaptable and equitable, to better support people with CP in overcoming barriers to dental services.

To our knowledge, this is the first Australian study to explore oral health among adults with CP using the OHIP-14 scale. While an important first step, limitations include its cross-sectional design, the partial inclusion of proxy reports, and the English-only online format that likely excluded non-English speaking households [35,36]. Respondents were predominantly adults with CP with more severe functional limitations (compared to those seen in the general CP population) from urban, advantaged areas; this may introduce potential selection bias and limit generalizability. A larger sample size is recommended to enable multivariate analysis. Rural–urban differences in service access were not explored; future research should include rural participants to better understand access barriers and examine potential differences between self- and proxy-reported OHIP-14 responses. While the OHIP-14 is widely used, some domain headings (e.g., “handicap”) can feel misaligned with their items; future adaptations could apply a strength-based lens for greater relevance [37]. Despite these limitations, the study highlights the need for accessible, individualized dental care for adults with CP. A follow-up qualitative study is underway to further explore dental service experiences for people with CP.

### Recommendations for Improving OHRQoL for Adults with CP

- Prioritize timely dental care for people with CP utilizing multidisciplinary and patient-centered approaches.
- Consider functional abilities (in particular MACS, CFCS, and VSS) when developing oral health care plans.
- Develop structured referral frameworks that promote equitable access to dental care for people with CP.
- Educate dental professionals in disability inclusive practices to enhance patient comfort and quality of care.
- Improve access to dental care, such as access to home and community-based care, mobile clinics, and telehealth, to enhance dental access for people with CP.
- Undertake further research to better understand the sociodemographic and geographical factors that affect access to dental care for people with CP. These findings should then be used to inform evidence-based policy, practices, and dental education programs.

## 5. Conclusions

In conclusion, this study highlights the OHRQoL and dental experiences of adults living with CP and their experiences participating in, accessing, and managing dental care. Physical, functional, and socio-economic factors impact OHRQoL. Barriers such as functional limitations, difficulty with undertaking routine oral hygiene practices, and inconsistent dental access emphasize the need for flexible, multidisciplinary, patient-centered, inclusive services.

## Figures and Tables

**Table 1:** Demographic information and clinical characteristics of adults with CP.

Variable		n	%
Age group	18–25 years	29	42.0
	26-35 years	17	24.6
	36-45 years	16	23.2
	46-55 years	4	5.8
	over 55 years	3	4.3
Gender	Males	35	50.7
	Females	34	49.3
Self-report		22	31.9
Proxy-report		47	68.1
Country of birth	Not stated	1	1.4
	Australia	65	94.2
	Overseas	3	4.3
Remoteness (Australia)	Not stated	2	2.9
	Major cities	52	75.4
	Inner regional	11	15.9
	Outer regional	4	5.8
SEIFA	Not stated	2	2.9
	Quintile 1	8	11.6
	Quintile 2	13	18.8
	Quintile 3	9	13.0
	Quintile 4	15	21.7
	Quintile 5	22	31.9
CP motor type	Not stated	11	15.9
	Ataxic	7	10.1
	Spastic	46	66.7
	Dyskinetic	5	7.2
	Unilateral	16	34.8
	Bilateral	30	65.2
GMFCS scale	Levels I-II	23	33.3
	Levels III-V	46	66.7
MACS scale	Levels I-II	20	29.0
	Levels III-V	49	71.0
VSS scale	Levels I-II	36	52.2
	Levels III-IV	33	47.8
CFCS	Levels I-II	46	66.7
	Levels III-IV	23	33.3
Hearing	Bilateral deafness	4	5.8
	No impairment	56	81.2
	Some impairment	9	13.0
Epilepsy	Yes	33	47.8
Anti-seizure medication	Yes	28	40.6

SEIFA: Socio-Economic Indexes for Areas [16], GMFCS: Gross Motor Function Classification System [17], MACS: Manual Ability Classification System [18], VSS: Viking Speech Scale [19], CFCS: Communication Function Classification System [20].

**Table 2:** Reliability analysis based on the corrected item–total correlation and Cronbach’s alpha coefficient if the item is deleted.

Scale item	Corrected Item-Total Correlation	Cronbach’s Alpha if Item Deleted
OHIP-14—Trouble pronouncing any words	0.599	0.896
OHIP-14—Worse sense of taste	0.591	0.895
OHIP-14—Painful aching in the mouth	0.387	0.902
OHIP-14—Uncomfortable to eat any foods	0.710	0.890
OHIP-14—Been self-conscious	0.642	0.893
OHIP-14—Felt tense	0.543	0.897
OHIP-14—Unsatisfactory diet	0.720	0.889
OHIP-14—Interrupted meals	0.735	0.889
OHIP-14—Difficult to relax	0.700	0.890
OHIP-14—Been embarrassed	0.530	0.897
OHIP-14—Been irritable with other people	0.618	0.894
OHIP-14—Difficulty doing usual jobs	0.425	0.901
OHIP-14—Felt that life in general was less satisfying	0.666	0.892
OHIP-14—Been totally unable to function	0.504	0.899

**Table 3:** Spearman’s rank correlation coefficients for the daily routine, the OHIP-14 dimensions, and the total OHIP-14 score.

Dimensions	Oral health daily routine			r_s_	*p*-value
	“Fairly straightforward” n = 21 Mean ± SD	“A bit of a challenge” n = 37 Mean ± SD	“Extremely difficult” n = 11 Mean ± SD		
Functional limitation	0.6 ± 0.8	1.7 ± 2.1	2.6 ± 2.1	0.358	0.003
Physical pain	1.4 ± 1.7	2.4 ± 1.8	3.0 ± 1.4	0.336	0.005
Psychological discomfort	1.1 ± 1.6	1.7 ± 2.0	1.8 ± 1.9	0.142	0.245
Physical disability	0.5 ± 0.9	1.4 ± 1.8	3.0 ± 2.2	0.375	0.002
Psychological disability	1.1 ± 1.5	1.4 ± 1.6	1.8 ± 1.5	0.327	0.006
Social disability	0.4 ± 0.9	1.1 ± 1.5	2.0 ± 2.1	0.327	0.006
Handicap	0.6 ± 0.9	1.2 ± 1.4	1.1 ± 1.4	0.171	0.159
OHIP-14	6.0 ± 6.6	11.3 ± 9.8	15.5 ± 9.5	0.360	0.002

**Table 4:** The relationship between the OHIP-14 dimensions and demographic and clinical variables.

Variable	n	OHIP-14 scale total score			Functional limitation dimension			Physical pain dimension			Psychological discomfort dimension			Physical disability dimension			Psychological disability dimension			Social disability dimension			Handicap dimension		
		Mean ± SD	Median	*p*-value	Mean ± SD	Median	*p*-value	Mean ± SD	Median	*p*-value	Mean ± SD	Median	*p*-value	Mean ± SD	Median	*p*-value	Mean ± SD	Median	*p*-value	Mean ± SD	Median	*p*-value	Mean ± SD	Median	*p*-value
Age group																									
18–25 years	29	11.0 ± 10.5	9.5	0.527 (KWH)	1.4 ± 1.5	1.5	0.081 (KWH)	2.0 ± 1.8	1.0	0.255 (KWH)	1.9 ± 2.0	2.0	0.064 (KWH)	1.7 ± 2.1	1.0	0.321 (KWH)	1.5 ± 1.8	1.0	0.517 (KWH)	0.4 ± 0.7	0.0	0.886 (KWH)	1.0 ± 1.4	0.0	0.183 (KWH)
26–35 years	17	10.5 ± 9.2	7.0		2.3 ± 2.5	2.0		2.6 ± 1.9	3.0		0.6 ± 0.7	0.0		1.5 ± 1.9	0.0		1.1 ± 1.2	1.0		0.9 ± 1.3	0.0		0.8 ± 1.4	0.0	
36–45 years	16	4.5 ± 5.2	6.5		0.5 ± 0.7	0.0		2.3 ± 1.7	2.0		1.7 ± 2.0	1.5		0.5 ± 0.7	0.0		1.3 ± 1.5	1.0		1.1 ± 1.7	0.0		0.9 ± 1.0	0.5	
46–55 years	4	4.5 ± 5.25	3.0		0.2 ± 0.5	0.0		1.2 ± 1.5	1.0		0.0 ± 0.0	0.0		0.7 ± 1.5	0.0		0.7 ± 0.9	0.5		0.7 ± 1.5	0.0		0.7 ± 0.9	0.5	
Over 55 years	3	16.0 ± 6.0	13.0		1.3 ± 0.5	1.0		4.0 ± 0.0	4.0		2.6 ± 2.5	3.0		1.6 ± 1.1	1.0		2.6 ± 1.1	2.0		1.0 ± 1.7	0.0		2.6 ± 0.5	3.0	
Gender																									
Males	35	10.6 ± 9.2	7.0	0.673 (MWU)	1.7 ± 1.7	2.0	0.100 (MWU)	2.1 ± 1.8	2.0	0.526 (MWU)	1.7 ± 1.8	2.0	0.299 (MWU)	1.4 ± 1.7	1.0	0.689 (MWU)	1.6 ± 1.7	1.0	0.438 (MWU)	0.9 ± 1.2	0.0	0.766 (MWU)	1.0 ± 1.3	0.0	0.942 (MWU)
Females	34	10.6 ± 9.6	7.0		1.3 ± 2.1	0.0		2.4 ± 1.8	2.0		1.4 ± 2.0	0.0		1.4 ± 2.0	0.0		1.2 ± 1.4	1.0		1.1 ± 1.8	0.0		1.0 ± 1.3	0.0	
Survey responses																									
Self-report	22	9.0 ± 7.7	7.0	0.557 (MWU)	0.6 ± 0.8	0.0	0.020 (MWU)	2.2 ± 1.7	2.0	0.999 (MWU)	1.6 ± 2.1	0.5	0.973 (MWU)	0.7 ± 1.1	0.0	0.086 (MWU)	1.6 ± 1.7	1.5	0.604 (MWU)	0.9 ± 1.5	0.0	0.486 (MWU)	1.0 ± 1.1	0.5	0.798 (MWU)
Proxy-report	47	11.0 ± 10.0	7.0		1.9 ± 2.1	1.0		2.2 ± 1.8	2.0		1.5 ± 1.8	1.0		1.7 ± 2.0	1.0		1.3 ± 1.5	1.0		1.1 ± 1.5	0.0		1.0 ± 1.4	0.0	
Remoteness (Australia)																									
Major Cities	52	10.6 ± 10.0	7.0	0.812 (KWH)	1.7 ± 2.1	1.0	0.251 (KWH)	2.2 ± 1.8	2.0	0.764 (KWH)	1.3 ± 1.8	0.0	0.180 (KWH)	1.5 ± 1.9	0.0	0.551 (KWH)	1.5 ± 1.6	1.0	0.560 (KWH)	1.1 ± 1.7	0.0	0.244 (KWH)	1.1 ± 1.7	0.0	0.493 (KWH)
Inner Regional	11	10.0 ± 8.1	8.0		0.6 ± 0.8	0.0		2.5 ± 1.8	3.0		2.4 ± 2.2	2.0		1.0 ± 1.3	1.0		1.4 ± 1.8	0.0		0.7 ± 1.1	0.0		0.7 ± 1.1	0.0	
Outer Regional	4	6.0 ± 4.0	6.0		0.7 ± 0.9	0.5		1.7 ± 1.7	1.5		2.0 ± 1.4	1.5		0.5 ± 1.0	0.0		0.5 ± 0.5	0.5		0 ± 0	0.0		0.0 ± 0.0	0.0	
SEIFA																									
Quintile 1	8	13.8 ± 8.3	12.0	0.373 (KWH)	2.1 ± 2.4	1.5	0.758 (KWH)	2.3 ± 2.0	1.5	0.945 (KWH)	2.3 ± 2.4	2.0	0.432 (KWH)	1.8 ± 1.6	2.0	0.459 (KWH)	2.6 ± 1.5	2.0	0.076 (KWH)	0.8 ± 1.3	0.0	0.977 (KWH)	0.8 ± 1.3	0.0	0.040 (KWH)
Quintile 2	13	8.3 ± 8.8	6.0		1.3 ± 1.9	0.0		2.2 ± 2.2	2.0		1.4 ± 1.6	1.0		1.2 ± 1.6	0.5		0.9 ± 1.6	0.0		0.6 ± 0.7	0.0		0.6 ± 0.7	0.0	
Quintile 3	9	13.2 ± 9.2	15.0		1.5 ± 1.8	1.0		2.6 ± 1.7	3.0		2.1 ± 2.2	2.0		1.8 ± 1.9	1.0		1.6 ± 1.5	2.0		1.3 ± 1.9	0.0		1.3 ± 1.9	0.0	
Quintile 4	15	10.3 ± 8.7	7.0		1.4 ± 1.5	1.0		2.3 ± 1.6	3.0		1.6 ± 2.0	1.0		1.3 ± 1.9	0.0		1.4 ± 1.4	1.0		1.2 ± 1.8	0.0		1.2 ± 1.8	0.0	
Quintile 5	22	8.8 ± 10.9	3.5		1.5 ± 2.1	0.0		2.0 ± 1.8	2.0		1.0 ± 1.7	0.0		1.1 ± 2.0	0.0		1.1 ± 1.6	0.0		1.0 ± 1.7	0.0		1.0 ± 1.7	0.0	
Type of CP																									
Not stated	11	10.9 ± 9.4	11.0	0.892 (KWH)	2.1 ± 2.3	1.0	0.634 (KWH)	1.9 ± 2.0	1.0	0.776 (KWH)	1.0 ± 1.4	0.0	0.623 (KWH)	2.1 ± 2.2	2.0	0.336 (KWH)	0.9 ± 0.9	1.0	0.877 (KWH)	1.1 ± 1.8	1.0	0.872 (KWH)	1.1 ± 1.8	1.0	0.821 (KWH)
Ataxic	7	7.5 ± 6.2	11.0		0.7 ± 0.9	0.0		1.8 ± 1.3	2.0		1.0 ± 1.2	0.0		0.4 ± 0.7	0.0		1.5 ± 1.8	2.0		1.0 ± 1.4	0.0		1.0 ± 1.4	0.0	
Dyskinetic	5	12.4 ± 15.6	2.0		2.4 ± 3.3	0.0		2.4 ± 1.5	2.0		2.0 ± 2.8	0.0		2.0 ± 3.0	0.0		1.8 ± 2.4	0.0		0.8 ± 1.7	0.0		0.8 ± 1.7	0.0	
Spastic	46	10.6 ± 9.2	7.0		1.4 ± 1.7	1.0		2.4 ± 1.8	2.0		1.7 ± 1.9	1.0		1.3 ± 1.6	0.5		1.4 ± 1.6	1.0		1.0 ± 1.5	0.0		1.0 ± 1.5	0.0	
*Unilateral*	16	10.8 ± 9.1	9.5	0.999 (MWU)	1.4 ± 2.0	1.0	0.800 (MWU)	2.0 ± 1.8	1.5	0.223 (MWU)	2.1 ± 2.5	1.0	0.773 (MWU)	1.2 ± 1.6	0.5	0.795 (MWU)	1.7 ± 1.4	2.0	0.291 (MWU)	0.8 ± 1.4	0.0	0.393 (MWU)	1.3 ± 1.3	2.0	0.282 (MWU)
*Bilateral*	30	10.5 ±9.3	7.0		1.4 ± 1.5	1.0		2.6 ± 1.8	2.5		1.5 ± 1.6	1.0		1.4 ± 1.3	0.5		1.3 ± 1.6	0.5		1.2 ± 1.6	0.0		0.9 ± 1.4	0.0	
GMFCS																									
Levels I-II	23	8.7 ± 9.0	6.0	0.199 (MWU)	1.0 ± 1.3	0.0	0.166 (MWU)	1.7 ± 1.9	1.0	0.037 (MWU)	1.6 ± 2.1	1.0	0.979 (MWU)	1.0 ± 1.5	0.0	0.324 (MWU)	1.3 ± 1.4	1.0	0.936 (MWU)	0.8 ± 1.5	0.0	0.430 (MWU)	0.8 ± 1.5	0.0	0.944 (MWU)
Levels III-V	46	11.1 ± 9.5	8.0		1.7 ± 2.1	1.0		2.5 ± 1.6	2.0		1.5 ± 1.8	1.0		1.6 ± 2.0	1.0		1.4 ± 1.6	1.0		1.1 ± 1.5	0.0		1.1 ± 1.5	0.0	
MACS																									
Levels I-II	18	3.7 ± 4.6	1.5	<0.001 (MWU)	0.8 ± 1.6	0.0	0.015 (MWU)	1.4 ± 1.7	1.0	0.009 (MWU)	0.7 ± 0.8	0.0	0.037 (MWU)	0.8 ± 1.5	0.0	0.058 (MWU)	0.6 ± 0.9	0.0	0.013 (MWU)	0.4 ± 0.8	0.0	0.053 (MWU)	0.4 ± 0.8	0.0	0.114 (MWU)
Levels III-V	49	12.2 ± 9.5	12.0		1.8 ± 1.9	1.0		2.6 ± 1.7	3.0		1.9 ± 2.1	2.0		1.6 ± 1.9	1.0		1.7 ± 1.6	2.0		1.3 ± 1.7	0.0		1.3 ± 1.7	0.0	
VSS																									
Levels I-II	36	8.4 ± 7.8	6.5	0.123 (MWU)	0.9 ± 1.1	0.0	0.024 (MWU)	2.0 ± 1.7	2.0	0.222 (MWU)	1.5 ± 1.9	1.0	0.909 (MWU)	0.9 ± 1.3	0.0	0.062 (MWU)	1.3 ± 1.5	0.5	0.472 (MWU)	0.8 ± 1.4	0.0	0.196 (MWU)	0.8 ± 1.4	0.0	0.439 (MWU)
Levels III-IV	33	12.4 ± 10.5	12.0		2.2 ± 2.3	1.0		2.5 ± 1.8	2.0		1.6 ± 1.9	1.0		1.9 ± 2.1	1.0		1.5 ± 1.6	1.0		1.3 ± 1.7	0.0		1.3 ± 1.7	0.0	
CFCS																									
Levels I-II	46	9.4 ± 8.6	6.5	0.319 (MWU)	1.1 ± 1.3	1.0	0.109 (MWU)	2.2 ± 1.7	2.0	0.826 (MWU)	1.6 ± 1.9	1.0	0.846 (MWU)	1.1 ± 1.6	0.0	0.033 (MWU)	1.4 ± 1.6	1.0	0.957 (MWU)	0.9 ± 1.5	0.0	0.322 (MWU)	0.9 ± 1.5	0.0	0.916 (MWU)
Levels III-V	23	12.2 ± 10.6	11.0		2.3 ± 2.6	1.0		2.3 ± 1.8	2.0		1.5 ± 1.9	1.0		2.1 ± 2.1	2.0		1.3 ± 1.5	1.0		1.3 ± 1.6	0.0		1.3 ± 1.6	0.0	
Hearing																									
No impairment	56	10.1 ± 9.6	7.0	0.714 (KWH)	1.4 ± 1.7	1.0	0.307 (KWH)	2.1 ± 1.8	2.0	0.465 (KWH)	1.6 ± 1.8	1.0	0.793 (KWH)	1.4 ± 1.8	0.0	0.646 (KWH)	1.3 ± 1.6	1.0	0.532 (KWH)	1.1 ± 1.6	0.0	0.657 (KWH)	1.1 ± 1.6	0.0	0.819 (KWH)
Some impairment	9	10.5 ± 9.1	11.0		1.5 ± 2.4	0.0		2.6 ± 2.0	3.0		1.4 ± 1.8	1.0		1.3 ± 1.8	0.0		1.4 ± 1.3	2.0		0.8 ± 1.1	0.0		0.8 ± 1.1	0.0	
Bilateral deafness	4	13.0 ± 8.2	14.5		3.2 ± 2.7	3.5		3.0 ± 1.1	3.0		1.5 ± 3.0	0.0		2.0 ± 1.8	2.0		2.2 ± 2.0	2.0		0.2 ± 0.5	0.0		0.2 ± 0.5	0.0	
Epilepsy																									
Yes	33	11.9 ± 10.3	11.0	0.252 (MWU)	2.1 ± 2.3	1.0	0.040 (MWU)	2.5 ± 1.8	2.0	0.308 (MWU)	1.4 ± 1.6	1.0	0.874 (MWU)	1.9 ± 2.2	1.0	0.129 (MWU)	1.3 ± 1.4	1.0	0.799 (MWU)	1.3 ± 1.7	0.0	0.123 (MWU)	1.3 ± 1.7	0.0	0.947 (MWU)
No	36	8.9 ± 8.2	7.0		1.0 ± 1.3	0.0		2.0 ± 1.7	2.0		1.7 ± 2.1	1.0		0.9 ± 1.2	0.0		1.4 ± 1.7	0.5		0.7 ± 1.2	0.0		0.7 ± 1.2	0.0	
Anti-seizure medication																									
Yes	28	13.2 ± 10.7	12.5	0.129 (MWU)	2.3 ± 2.3	2.0	0.104 (MWU)	2.5 ± 1.9	2.0	0.981 (MWU)	1.5 ± 1.6	1.0	0.715 (MWU)	2.3 ± 2.2	2.5	0.030 (MWU)	1.6 ± 1.4	2.0	0.034 (MWU)	1.6 ± 1.8	1.0	0.045 (MWU)	1.6 ± 1.8	1.0	0.058 (MWU)
No	5	4.4 ± 2.8	6.0		0.6 ± 0.8	0.0		2.6 ± 1.6	3.0		1.0 ± 1.0	1.0		0.0 ± 0.0	0.0		0.2 ± 0.4	0.0		0.0 ± 0.0	0.0		0.0 ± 0.0	0.0	

“Mann-Whitney” U test (MWU), “Kruskal-Wallis” H Test (KWH).

**Table 5:** Experience of dental care and services.

Variable	n	%
Is your daily routine of teeth cleaning?		
Fairly straightforward	21	30.4
A bit of a challenge	37	53.6
Extremely difficult	11	15.9
Do you have a regular dentist?		
My whole family uses the same dentist.	33	47.8
I have my own dentist or more than one dentist.	13	18.8
Different family members in my family have different dentists.	5	7.2
I do not have a dentist.	18	26.1
Does cerebral palsy affect your capacity to participate in dental examinations and treatment?		
Yes	44	63.8
If you have previously accessed dental treatment, e.g., exam, filling, cap/crown etc. Please select all that apply.		
Simple dental check-up clean, no special requirements needed.	33	47.8
Special arrangements were needed to attend the local practice	17	23.5
Some sedation was needed at the general dentist.	8	11.6
Dental treatment needed to be performed at a day surgery/hospital.	25	36.2
Never accessed dental treatment.	3	4.3
Have you ever had a dental professional (including Dental Hygienist, Dental Therapist, Oral Health Therapist or Dentist) come to your home for examination or treatment?		
Yes	2	2.9
Have you had to find urgent dental care due to pain or another problem(s)?		
Yes	17	24.6
Have you required complex dental surgery? On what basis has the complex surgery been provided?		
Emergency	3	4.3
Planned	22	31.9
Emergency and planned	4	5.8
Not applicable	40	58.0
If you needed complex dental surgery, how were the arrangements made?		
I made the arrangements	21	30.4
I was given assistance by another medical/dental practitioner.	9	13.0
Not applicable	39	56.5

**Table 6:** The relationship between OHIP-14 dimensions and experiences of dental care and services.

Variable	n	OHIP-14 scale total score			Functional limitation dimension			Physical pain dimension			Psychological discomfort dimension			Physical disability dimension			Psychological disability dimension			Social disability dimension			Handicap dimension		
		Mean ± SD	Median	*p*-value	Mean ± SD	Median	*p*-value	Mean ± SD	Median	*p*-value	Mean ± SD	Median	*p*-value	Mean ± SD	Median	*p*-value	Mean ± SD	Median	*p*-value	Mean ± SD	Median	*p*-value	Mean ± SD	Median	*p*-value
Is your daily routine of teeth cleaning?																									
Fairly straightforward	21	6.0 ± 6.6	4.0	0.012 (KWH)	0.6 ± 0.8	0.0	0.013 (KWH)	1.4 ± 1.7	1.0	0.021 (KWH)	1.1 ± 1.6	0.0	0.482 (KWH)	0.5 ± 0.9	0.0	0.007 (KWH)	1.1 ± 1.5	0.0	0.297 (KWH)	0.4 ± 0.9	0.0	0.026 (KWH)	0.6 ± 0.9	0.0	0.302 (KWH)
A bit of a challenge	37	11.3 ± 9.8	8.0		1.7 ± 2.1	1.0		2.4 ± 1.8	2.0		1.7 ± 2.0	1.0		1.4 ± 1.8	1.0		1.4 ± 1.6	1.0		1.1 ± 1.5	0.0		1.2 ± 1.4	0.0	
Extremely difficult	11	15.5 ± 9.5	16.0		2.6 ± 2.1	2.0		3.0 ± 1.4	3.0		1.8 ± 1.9	2.0		3.0 ± 2.2	3.0		1.8 ± 1.5	2.0		2.0 ± 2.1	1.0		1.1 ± 1.4	1.0	
Do you have a regular dentist?																									
My whole family uses the same dentist.	13	9.3 ± 9.5	6.0	0.784 (KWH)	1.5 ± 1.6	1.0	0.885 (KWH)	1.8 ± 1.9	2.0	0.317 (KWH)	1.5 ± 1.9	1.0	0.542 (KWH)	1.2 ± 1.7	0.0	0.885 (KWH)	1.3 ± 1.6	1.0	0.984 (KWH)	1.0 ± 1.7	1.0	0.932 (KWH)	0.7 ± 1.3	0.0	0.651 (KWH)
I have my own dentist or more than one dentist.	33	9.9 ± 9.6	6.0		1.3 ± 1.8	1.0		2.0 ± 1.8	1.0		1.3 ± 1.8	0.0		1.4 ± 1.9	1.0		1.3 ± 1.5	1.0		1.1 ± 1.6	0.0		1.1 ± 1.3	1.0	
Different family members in my family have different dentists.	5	12.6 ± 9.6	13.0		2.0 ± 2.3	1.0		3.2 ± 1.9	4.0		2.0 ± 1.8	2.0		1.2 ± 2.1	0.0		1.6 ± 1.6	2.0		1.0 ± 1.4	0.0		1.6 ± 2.0	1.0	
I do not have a dentist.	18	11.2 ± 9.2	9.0		1.6 ± 2.3	0.5		2.6 ± 1.5	2.0		1.9 ± 2.0	2.0		1.6 ± 1.9	0.5		1.5 ± 1.7	1.0		0.9 ± 1.4	0.0		0.8 ± 1.1	0.0	
Does cerebral palsy affect your capacity to participate in dental examinations and treatment?																									
Yes	44	11.1 ± 10.1	7.5	0.511 (MWU)	1.9 ± 2.1	1.5	0.069 (MWU)	2.4 ± 1.7	2.0	0.284 (MWU)	1.6 ± 1.9	1.0	0.927 (MWU)	1.6 ± 2.0	1.0	0.320 (MWU)	1.4 ± 1.5	1.0	0.884 (MWU)	1.1 ± 1.7	0.0	0.910 (MWU)	0.9 ± 1.4	0.0	0.271 (MWU)
No	25	9.0 ± 7.9	7.0		0.8 ± 1.1	0.0		2.0 ± 1.8	2.0		1.5 ± 1.9	1.0		1.0 ± 1.3	0.0		1.4 ± 1.6	1.0		0.9 ± 1.2	0.0		1.1 ± 1.2	1.0	
If you have previously accessed dental treatment, e.g., exam, filling, cap/crown etc. Please select all that apply.																									
Simple dental check-up clean, no special requirements needed.																									
Yes	33	7.7 ± 8.5	4.0	0.017 (MWU)	0.8 ± 1.2	0.0	0.007 (MWU)	1.8 ± 1.7	1.0	0.039 (MWU)	1.3 ± 1.9	0.0	0.179 (MWU)	0.8 ± 1.4	0.0	0.006 (MWU)	1.3 ± 1.7	0.0	0.343 (MWU)	0.8 ± 1.4	0.0	0.138 (MWU)	0.7 ± 1.1	0.0	0.090 (MWU)
No	36	12.7 ± 9.6	11.5		2.1 ± 2.2	1.5		2.6 ± 1.8	2.0		1.7 ± 1.8	1.5		2.0 ± 2.0	2.0		1.5 ± 1.5	1.5		1.2 ± 1.6	0.5		1.2 ± 1.4	1.0	
Special arrangements were needed to attend the local practice																									
Yes	17	7.8 ± 9.6	4.0	0.144 (MWU)	1.0 ± 1.6	0.0	0.174 (MWU)	2.1 ± 1.8	2.0	0.810 (MWU)	1.2 ± 1.8	0.0	0.329 (MWU)	1.0 ± 1.7	0.0	0.182 (MWU)	1.1 ± 1.6	0.0	0.244 (MWU)	0.7 ± 1.6	0.0	0.180 (MWU)	0.4 ± 1.0	0.0	0.034 (MWU)
No	52	11.1 ± 9.2	11.0		1.6 ± 2.0	1.0		2.3 ± 1.8	2.0		1.7 ± 1.9	1.0		1.5 ± 1.8	1.0		1.5 ± 1.5	1.5		1.1 ± 1.5	0.0		1.2 ± 1.3	1.0	
Some sedation was needed at the general dentist.																									
Yes	8	11.3 ± 10.6	9.5	0.807 (MWU)	1.6 ± 2.1	1.0	0.976 (MWU)	2.6 ± 1.6	2.5	0.505 (MWU)	1.5 ± 2.0	0.5	0.812 (MWU)	1.7 ± 2.1	0.5	0.664 (MWU)	1.7 ± 1.6	2.0	0.512 (MWU)	0.8 ± 1.4	0.0	0.792 (MWU)	1.2 ± 1.7	0.5	0.796 (MWU)
No	61	10.2 ± 9.3	7.0		1.5 ± 1.9	1.0		2.2 ± 1.8	2.0		1.6 ± 1.9	1.5		1.4 ± 1.8	0.0		1.3 ± 1.5	1.0		1.0 ± 1.5	1.0		1.0 ± 1.3	0.0	
Dental treatment needed to be performed at a day surgery/hospital.																									
Yes	25	11.6 ± 9.9	7.0	0.213 (MWU)	2.0 ± 1.9	2.0	0.052 (MWU)	2.6 ± 1.5	2.0	0.161 (MWU)	2.0 ± 1.9	2.0	0.096 (MWU)	1.6 ± 2.1	1.0	0.502 (MWU)	1.5 ± 1.6	1.0	0.526 (MWU)	1.0 ± 1.8	0.0	0.482 (MWU)	1.0 ± 1.3	0.0	0.978 (MWU)
No	44	9.4 ± 9.0	7.5		1.2 ± 1.8	0.0		2.0 ± 1.9	2.0		1.3 ± 1.8	0.0		1.3 ± 1.7	0.0		1.3 ± 1.5	0.5		1.0 ± 1.4	0.0		1.0 ± 1.3	0.0	
Never accessed dental treatment.																									
Yes	3	10.0 ± 16.4	1.0	0.585 (MWU)	2.6 ± 4.6	0.0	0.901 (MWU)	2.3 ± 3.2	1.0	0.857 (MWU)	2.0 ± 3.4	0.0	0.879 (MWU)	1.6 ± 2.8	0.0	0.945 (MWU)	1.3 ± 2.3	0.0	0.771 (MWU)	0.0 ± 0.0	0.0	0.235 (MWU)	0.0 ± 0.0	0.0	0.181 (MWU)
No	66	10.3 ± 9.1	7.5		1.4 ± 1.7	1.0		2.2 ± 1.7	2.0		1.5 ± 1.8	1.0		1.4 ± 1.8	0.5		1.4 ± 1.5	1.0		1.1 ± 1.5	0.0		1.0 ± 1.3	0.0	
Have you ever had a dental professional (including Dental Hygienist, Dental Therapist, Oral Health Therapist or Dentist) come to your home for examination or treatment?																									
Yes	2	12.5 ± 17.6	12.5	0.986 (MWU)	1.0 ± 1.4	1.0	0.819 (MWU)	1.5 ± 2.1	1.5	0.554 (MWU)	3.0 ± 4.2	3.0	0.668 (MWU)	1.0 ± 1.4	1.0	0.846 (MWU)	2.0 ± 2.8	2.0	0.793 (MWU)	2.5 ± 3.5	2.5	0.533 (MWU)	1.5 ± 2.1	1.5	0.692 (MWU)
No	67	10.3 ± 9.2	7.0		1.5 ± 1.9	1.0		2.3 ± 1.8	2.0		1.5 ± 1.8	1.0		1.4 ± 1.8	0.0		1.4 ± 1.5	1.0		1.0 ± 1.5	0.0		1.0 ± 1.3	0.0	
Have you had to find urgent dental care due to pain or another problem(s)?																									
Yes	17	10.2 ± 8.5	11.0	0.889 (MWU)	1.4 ± 1.8	1.0	0.872 (MWU)	2.2 ± 1.7	2.0	0.932 (MWU)	1.8 ± 1.9	1.0	0.463 (MWU)	1.3 ± 1.6	1.0	0.782 (MWU)	1.4 ± 1.6	1.0	0.836 (MWU)	0.8 ± 1.4	0.0	0.371 (MWU)	1.1 ± 1.5	0.0	0.713 (MWU)
No	52	10.3 ± 9.7	7.0		1.5 ± 1.9	1.0		2.2 ± 1.8	2.0		1.5 ± 1.9	1.0		1.4 ± 1.9	0.0		1.4 ± 1.6	1.0		1.1 ± 1.6	0.0		0.9 ± 1.3	0.0	
Have you required complex dental surgery? On what basis has the complex surgery been provided?																									
Emergency	3	10.6 ± 12.8	7.0	0.971 (KWH)	1.6 ± 1.5	2.0	0.589 (KWH)	1.6 ± 2.0	1.0	0.775 (KWH)	2.0 ± 2.0	2.0	0.783 (KWH)	1.6 ± 2.0	1.0	0.578 (KWH)	1.6 ± 2.0	1.0	0.977 (KWH)	1.3 ± 2.3	0.0	0.129 (KWH)	0.6 ± 1.1	0.0	0.585 (KWH)
Planned	22	10.7 ± 10.1	6.5		2.0 ± 2.3	1.0		2.5 ± 1.8	2.0		1.9 ± 2.2	1.0		1.3 ± 1.9	0.5		1.4 ± 1.6	1.0		0.6 ± 1.5	0.0		0.9 ± 1.4	0.0	
Emergency and planned	4	6.5 ± 4.7	5.5		0.7 ± 0.9	0.5		2.5 ± 0.5	2.5		0.7 ± 0.9	0.5		0.2 ± 0.5	0.0		1.0 ± 1.1	1.0		0.7 ± 1.5	0.0		0.5 ± 1.0	0.0	
Not applicable	40	10.5 ± 9.3	9.5		1.3 ± 1.7	0.5		2.1 ± 1.8	2.0		1.4 ± 1.8	1.0		1.6 ± 1.8	0.5		1.4 ± 1.5	1.5		1.3 ± 1.5	1.0		1.1 ± 1.3	1.0	
If you needed complex dental surgery, how were the arrangements made?																									
I made the arrangements	21	8.3 ± 9.6	6.0	0.104 (KWH)	1.5 ± 2.0	1.0	0.178 (KWH)	1.9 ± 1.6	2.0	0.077 (KWH)	1.2 ± 1.6	1.0	0.178 (KWH)	1.1 ± 1.9	0.0	0.164 (KWH)	1.1 ± 1.5	1.0	0.304 (KWH)	0.7 ± 1.5	0.0	0.689 (KWH)	0.6 ± 1.3	0.0	0.193 (KWH)
I was given assistance by another medical/dental practitioner.	9	13.3 ± 9.2	12.0		2.2 ± 2.2	2.0		3.2 ± 1.7	3.0		2.7 ± 2.5	3.0		1.4 ± 1.5	1.0		1.8 ± 1.7	2.0		0.5 ± 1.6	0.0		1.2 ± 1.3	1.0	
Not applicable	39	10.7 ± 9.2	11.0		1.3 ± 1.7	1.0		2.2 ± 1.8	2.0		1.5 ± 1.8	1.0		1.6 ± 1.8	1.0		1.4 ± 1.6	2.0		1.3 ± 1.5	1.0		1.2 ± 1.3	1.0	

“Mann-Whitney” U test (MWU), “Kruskal-Wallis” H Test (KWH).

**Table 7:** WLS regression coefficients and standard errors.

OHIP-14 score	B	SE B	95.0% CI for B		β	*p*-value	R^2^	ΔR^2^
			LL	UL				
Model							0.326	0.248
Constant	13.015	3.768	5.480	20.550		0.001		
Oral health daily routine (= “fairly straightforward”)	−5.855	3.628	−13.110	1.401	−0.354	0.112		
Oral health daily routine (= “a bit of a challenge”)	−3.115	3.373	−9.859	3.629	−0.181	0.359		
Oral health daily routine (= “extremely difficult”)	(reference)							
Gross Motor Function Classification System (GMFCS)	0.896	2.037	−3.177	4.969	0.054	0.662		
Manual Ability Classification system (MACS)	5.412	2.331	0.750	10.073	0.328	0.024		
Viking Speech Scale (VSS)	−1.908	2.677	−7.261	3.444	−0.099	0.479		
Simple dental check-up clean, no special requirements needed	−3.600	2.024	−7.647	0.447	−0.213	0.080		
Special arrangements were needed to attend the local practice	−4.051	2.240	−8.531	0.428	−0.201	0.075		

B = unstandardized regression coefficient; CI = confidence interval; LL = lower limit; UL = upper limit; SE B = standard error of the coefficient; β = standardized coefficient; R^2^ = coefficient of determination; ΔR^2^ = adjusted R^2^.

## Data Availability

The data presented in this study are available on request from the corresponding author. The data are not publicly available due to ethical restrictions.

## Abbreviations

The following abbreviations are used in this manuscript:

OHRQoL	Oral Health-Related Quality of Life
CP	Cerebral Palsy
OHIP-14	Oral Health Impact Profile-14
REDCap	Research Electronic Data Capture
SEIFA	Socio-Economic Indexes for Areas
GMFCS	Gross Motor Function Classification System
MACS	Manual Ability Classification System
VSS	Viking Speech Scale
CFCS	Communication Function Classification System
MWU	Mann–Whitney U test
KWH	Kruskal–Wallis H test
CROSS	Consensus-Based Checklist for Reporting of Survey Studies
OHIP	Oral Health Impact
